# Supplementary material for: Impact of SARS-CoV-2 vaccination of children ages 5–11 years on COVID-19 disease burden and resilience to new variants in the United States, November 2021-March 2022: a multi-model study
Source: medRxiv. 2022 Mar 10:2022.03.08.22271905. Preprint. [Version 1] doi: 10.1101/2022.03.08.22271905 (PMC8936106; doi:10.1101/2022.03.08.22271905)
Supplement: 1 [file NIHPP2022.03.08.22271905V1-supplement-1.pdf]

medRxiv preprint doi: https://doi.org/10.1101/2022.03.08.22271905; this version posted March 10, 2022. The copyright holder for this preprint (which was not certified by peer review) is the author/funder, who has granted medRxiv a license to display the preprint in perpetuity.
It is made available under a CC-BY 4.0 International license .

Supplemental Methods:

COVID-19 Scenario Modeling Hub Round 9 Participating Teams:

- Columbia University (New York, NY) – Age-Stratified Model (CU-AGE-ST)
- Johns Hopkins University (Baltimore, MD) ID Dynamics COVID-19 Working Group (JHU_IDD-CovidSP)
- Johns Hopkins University Applied Physics Lab (Laurel, MD) - Bucky (JHUAPL-Bucky)
- Northeastern University (Boston, MA) MOBS Lab – GLEAM COVID (MOBS_NEU-GLEAM-COVID)
- University of North Carolina at Charlotte (Charlotte, NC) – hierbin (UNCC-hierbin)
- University of Notre Dame (Notre Dame, IN) – FRED (NotreDame-FRED)
- University of Southern California (Los Angeles, CA) - SIkJalpha (USC-SIkJalpha)
- University of Virginia (Charlottesville, VA) – adaptive (UVA-adaptive)
- University of Virginia (Charlottesville, VA) – EpiHiper (UVA-EpiHiper)

Projections

Probabilistic projections were collected using 23 quantiles (i.e., 0.01, 0.025, 0.05, every 5% to 0.95, 0.975, and 0.99).

Five teams did not include waning (CU-AGE-ST, JHUAPL-Bucky, JHU_IDD-CovidSP, UNCC-hierbin, NotreDame-FRED, see above list of participating teams for full team names). Three teams assumed that immunity would wane exponentially with an average period of either six months (UVA-EpiHiper) or one year (MOBS_NEU-GLEAM-COVID, UVA-adaptive). The USC-SIkJalpha team assumed a weighted average of assumptions about waning.

Five teams also submitted projections with the same quantile structure for a younger age-group (0-11, 5-11, 5-17, and 0-17 year groups) and an older age-group when possible:

- CU-AGE-ST
- MOBS_NEU-GLEAM_COVID
- USC-SIkJalpha
- UVA-adaptive
- UVA-EpiHiper

For scenarios without the emergence of a more transmissible variant, mean reported case reductions in younger age-groups ranged from 6.5 to 34.5% across models over the November 1, 2021 to March 12, 2022 time period. Reductions in hospitalizations and deaths ranged from 5.6 to 34.5% and 5.2 to 34.5%, respectively. For scenarios with the emergence of a more transmissible variant, mean projected reductions in cases were 10.9 to 32.9%, with hospitalization and death reductions ranging from 9.0 to 32.9% and 5.9 to 33.1% respectively. Percent reductions were not estimated for one team, which reported zero deaths for the younger age-group over the November 1, 2021 to March 12, 2022 time period.

Meta analyses

medRxiv preprint doi: https://doi.org/10.1101/2022.03.08.22271905; this version posted March 10, 2022. The copyright holder for this preprint (which was not certified by peer review) is the author/funder, who has granted medRxiv a license to display the preprint in perpetuity.
It is made available under a CC-BY 4.0 International license .

To summarize the projected benefits of the vaccine program expansion across all modeling teams, we used a standard meta-analytic approach with random effects[22]. We estimated the mean difference in cumulative incidence and the mean incidence ratio between scenarios with and without children 5-11 years old vaccinated, stratified by presence or absence of the new variant, for the portion of the projection period following the assumed start date of childhood vaccination (i.e., from November 1, 2021).

Specifically, for each scenario $s = A, B, C, D$, the modeling teams $m = 1,2,3 \ldots 9$ directly provided us with the the mean and variance (over their individual model replicates) of each cumulative outcome $o = Cases, Hospitalizations, Deaths,$ at the start of the vaccination period (t_0) and the end of the of projection period (t_1) at the national level. We then estimated the mean (μ) and variance (σ^2) for each model, scenario, and cumulative incidences over the period of interest as $\mu_{mso} = \mu_{mso}(t_1) - \mu_{mso}(t_0)$ and $\sigma^2_{mso} = \sigma^2_{mso}(t_1) + \sigma^2_{mso}(t_0)$, respectively. To compare scenarios, for example A (with vaccination of children 5-11 years old) and B (without vaccination of children 5-11 years old), we then estimated the mean of the difference as $\mu_{mAo} - \mu_{mBo}$ and the variance of the difference as $\sigma^2_{mAo} + \sigma^2_{mBo}$. We also estimated the incidence ratio as the ratio of the above means (e.g. $\frac{\mu_{mAo}}{\mu_{mBo}}$, with the proportion reduction due to the vaccine estimated as 1-$\frac{\mu_{mAo}}{\mu_{mBo}}$); the variance of this ratio was obtained using the delta method. For both absolute difference and incidence ratio, we estimated the standard error (SE) as $\sqrt{\sigma^2_*/n}$, where σ^2_* is the variance as defined above, and n is the number of replicates (simulations or sets of projected outcomes) for that model. Model specific means and standard errors were combined via random effects meta-analysis using restricted maximum likelihood (REML).

At the state level, individual modeling teams provided quantile distributions, as specified above, but did not provide us with model-specific estimates of mean and variance over their replicates at the two above time points of interest. Therefore, to evaluate vaccine benefits at the state level, we estimated the mean and variance from the 23 available points from each model-specific cumulative distribution function (CDF) at t_0 and t_1(again, for each outcome, scenario, and state). Specifically, to each CDF (n~11,000; 51 states x 9 models x 3 outcomes x 4 scenarios x 2 timepoints) we fit a penalized cubic spline Poisson regression model to estimate a continuous quantile function, from which we simulated 25,000 replicates. The mean and variance of these replicates were then estimated, and the above-described approaches were followed to obtain mean and variance for the absolute difference and incidence ratio between scenarios, and for combining these using random effects REML meta analyses. This procedure was also followed for approximating the direct effect of vaccine expansion (within the younger age group).

medRxiv preprint doi: https://doi.org/10.1101/2022.03.08.22271905; this version posted March 10, 2022. The copyright holder for this preprint (which was not certified by peer review) is the author/funder, who has granted medRxiv a license to display the preprint in perpetuity.
It is made available under a CC-BY 4.0 International license .

Supplemental Figures/Tables:

Figure S1: Model-specific trajectories for cases, hospitalizations, and deaths in each of the four scenarios. Differences between models were generally most apparent in the new variant scenarios. USC-SIkJalpha projected a large peak in cases in early 2022 in the variant scenarios. This peak was followed by peaks in hospitalizations and deaths. NEU-MOBs projected less of an impact in variant scenarios, with deep troughs observed at the national-level in early 2022. UVA-EpiHiper projected sharp decreases at the end of December 2021 and resurgences in the post-holiday period, driven by assumptions about school closures.

medRxiv preprint doi: https://doi.org/10.1101/2022.03.08.22271905; this version posted March 10, 2022. The copyright holder for this preprint (which was not certified by peer review) is the author/funder, who has granted medRxiv a license to display the preprint in perpetuity.
It is made available under a CC-BY 4.0 International license .

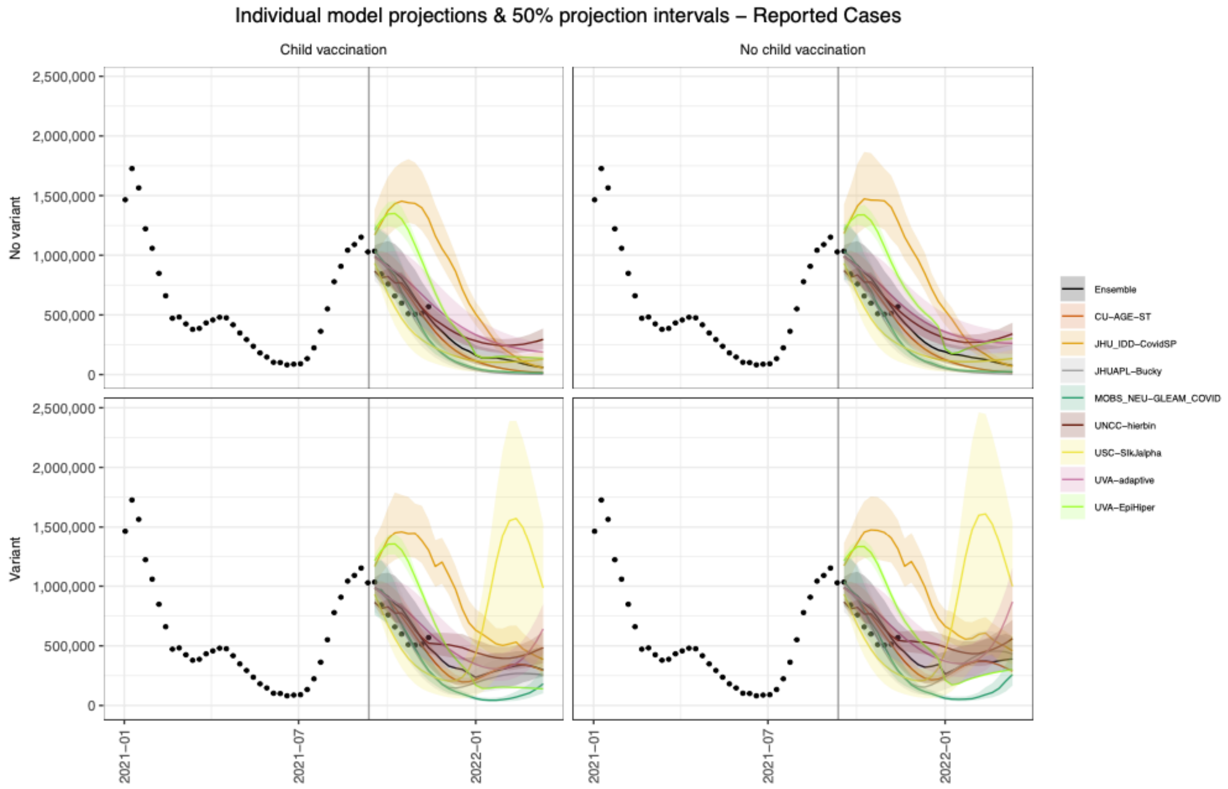

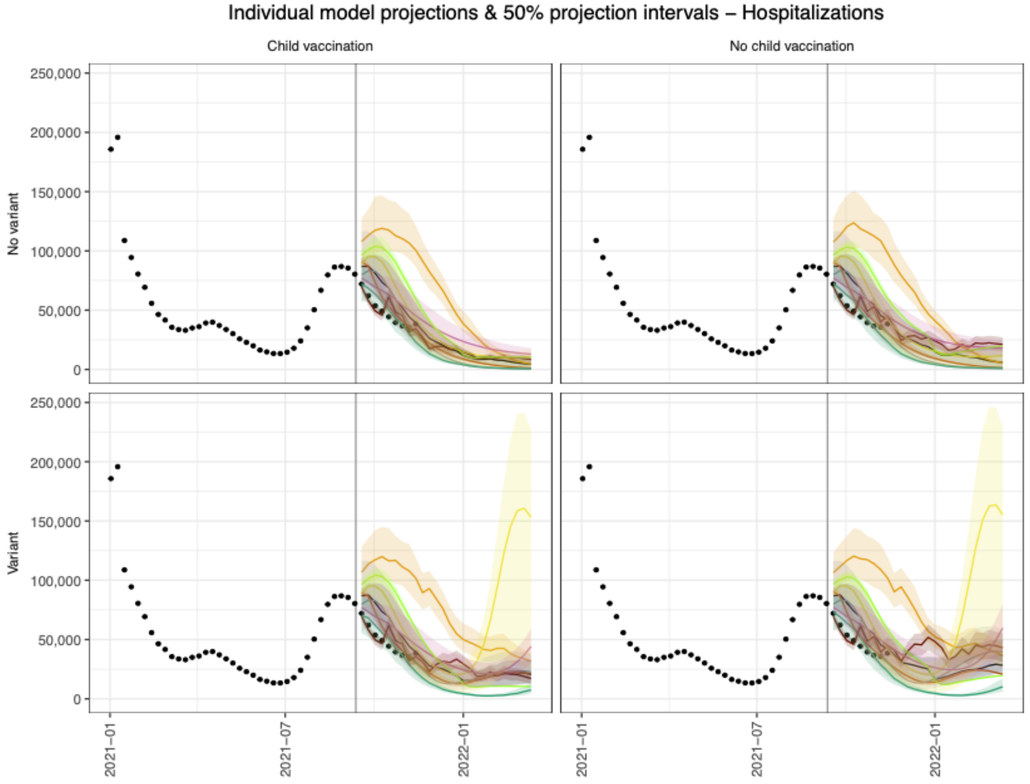

medRxiv preprint doi: https://doi.org/10.1101/2022.03.08.22271905; this version posted March 10, 2022. The copyright holder for this preprint (which was not certified by peer review) is the author/funder, who has granted medRxiv a license to display the preprint in perpetuity.
It is made available under a CC-BY 4.0 International license .

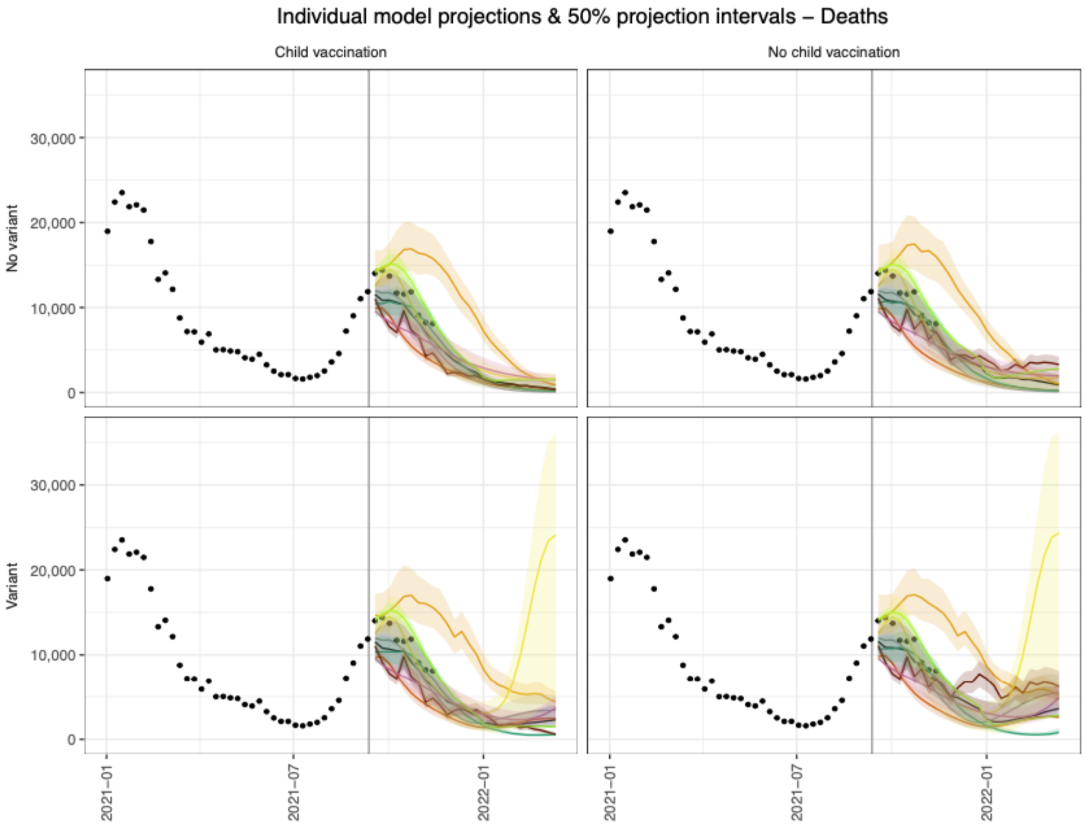

medRxiv preprint doi: https://doi.org/10.1101/2022.03.08.22271905; this version posted March 10, 2022. The copyright holder for this preprint (which was not certified by peer review) is the author/funder, who has granted medRxiv a license to display the preprint in perpetuity.
It is made available under a CC-BY 4.0 International license .

Figure S2: State-level estimates for number of vaccinated 5-11 year olds per capita vs. projected cases occurring between November 1, 2021 and March 12, 2022. Estimates for vaccinated 5-11 year olds are based on the vaccination coverage of 12-17 year olds on September 11, 2021 (the last day of data available for use in projections).

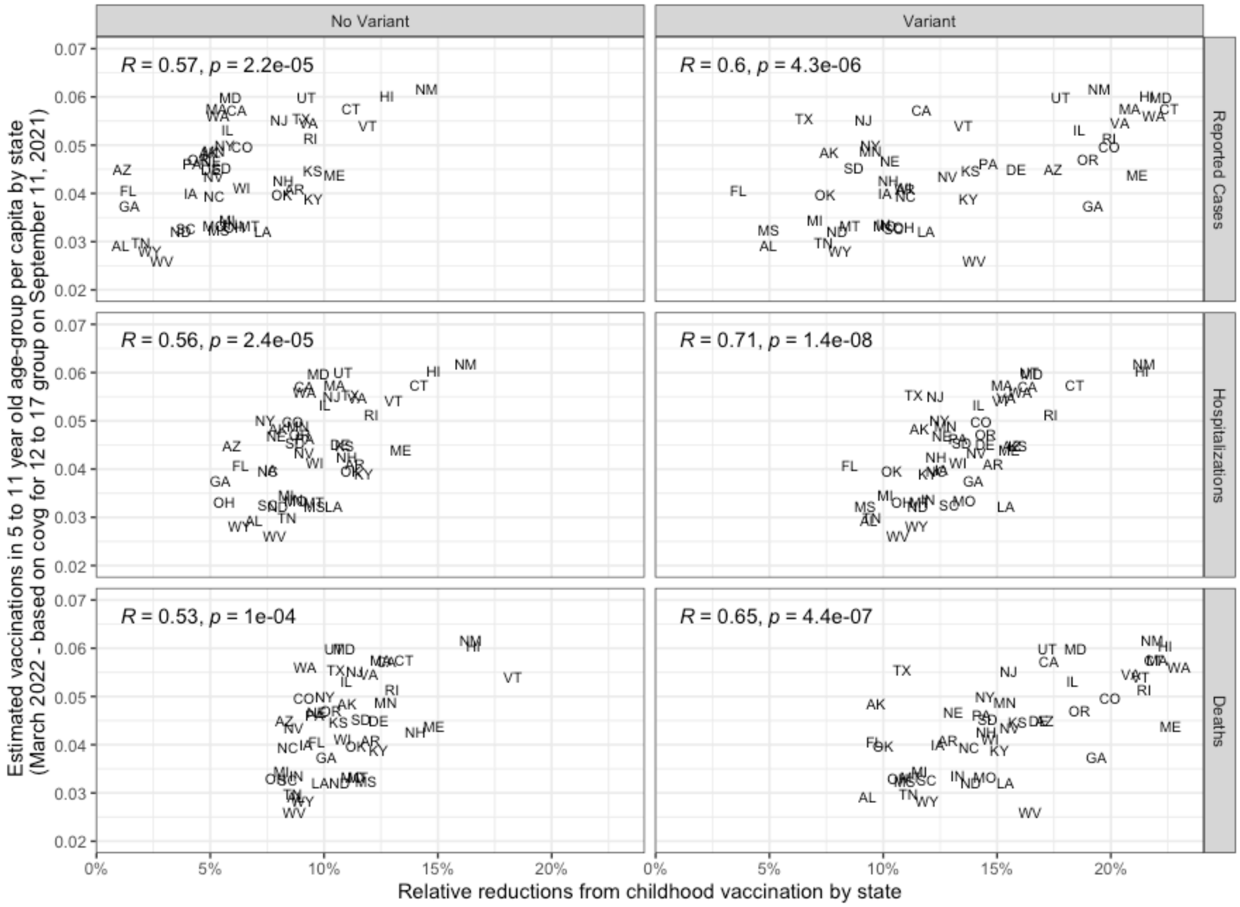

medRxiv preprint doi: https://doi.org/10.1101/2022.03.08.22271905; this version posted March 10, 2022. The copyright holder for this preprint
(which was not certified by peer review) is the author/funder, who has granted medRxiv a license to display the preprint in perpetuity.
It is made available under a CC-BY 4.0 International license .

Table S1: model-specific vaccine effectiveness (VE) assumptions.

Team	VE against infection	VE against symptoms	VE against hospitalization
CU-AGE-ST	30% 2 wks after 1st dose, 70% 2 wks after 2nd dose	30% 2 wks 1st dose, 70% 2 wks after 2nd dose	90% 2 wks after 2nd dose
JHU-APL-Bucky	means of 35% after 1st dose, 85% after 2nd dose (drawn from a distribution)	same as VE against infection	same as VE against infection
JHU_IDD-CovidSP	35% 2wks after 1st dose, 60% 2wks after second	35% 2wks after 1st dose, 60% 2wks after second	80% 2wks after second dose
NotreDame-FRED	non-Delta: 76%, Delta: 72%	non-Delta: 90%, Delta: 85%	non-Delta: 96%, Delta: 96%
MOBS-NEU-GLEAM_COVID	32% 2wks after 1st dose, 60% 2wks after 2nd dose	35% 2wks after 1st dose, 85% 2wks after 2nd dose	same as VE against symptoms
UNCC-Hierbin	60%-85% for all age groups and virus variants	same as VE against infection	90% for all age groups and virus variants
USC-SlkJalpha	85% minus waning	85% minus waning	(100% minus waning) if in immune state
UVA-Adaptive	same as VE against symptoms	50% after after 1st dose, 95% after second, 67% after J&J dose, minus waning	same as VE against symptoms
UVA-EpiHiper	same as VE against symptoms	35% after after 1st dose, 85% after second, 60% after J&J dose	same as VE against symptoms

Table S2: additional model-specific assumptions including those about nonpharmaceutical interventions (NPIs).

	**CU-AGE-ST**	**JHUAPL_Bucky**	**JHU_IDD-CovidSP**	**NotreDame-FRED**	**MOBS_NEU-GLEAM_COVID**	**UNCC-hierbin**	**USC-SlkJalpha**	**UVA-adaptive**	**UVA-EpiHiper**
**Model Type**	Compartmental	Meta-population compartmental	Meta-population compartmental	Agent-based	Meta-population compartmental	Trajectory tracking (non-mechanistic)	Discrete time heterogeneous rate compartmental	Meta-population compartmental	Agent-based
**Geography**	County	County	State	State	County	State	State	County	State
**Mobility and contact data**	SafeGraph mobility, POLYMOD contact rates	SafeGraph mobility, age-based contact matrices	Commuting	Google mobility	Google mobility, commuting, flight, age-based contact matrices	Not used	Cuebiq contact scores data to model future NPI changes	Not used	ACS Commute, National Household Travel Survey

medRxiv preprint doi: https://doi.org/10.1101/2022.03.08.22271905; this version posted March 10, 2022. The copyright holder for this preprint (which was not certified by peer review) is the author/funder, who has granted medRxiv a license to display the preprint in perpetuity.
It is made available under a CC-BY 4.0 International license .

**Age groups**	5	16	Age-adjusted for vacc, hosp, and death	Individual ages	10	None	10	Hosp and death outcomes adjusted for 3 age groups	5
**Waning of natural immunity**	None	None	None	None	None	None	None	None	None
**Wanning of vaccine immunity**	None	None	None	None	Leaky vaccine	None	Leaky vaccine	None	None
**Importations**	No	No	No	Low constant importations per variant	Domestic/International	None	No	Low steady importation per county, 1 per million population	None
**Multiple strains**	Proportion of variant cases by time imported from simplified two-strain model	Proportion of variant cases estimated from simplified model	No	Co-circulating variants matching dominance data.	2 strains	No	Separate model for each strain	No	Two strains
**NPI history**	Fitted transmissibility; no additional NPI relaxation	Fitted based on transmission rates	Fitted based on state-level reported periods of changing policy	Fitted based on historic deaths	Mobility + Oxford data	Based on historical data	Cuebiq data to assess current contact scores	Modeled by proxy using fitted transmissibility	Not modeled
**NPI relaxation**	Maximum relaxation reached	Linear	Linear	NPIs relaxed for the projection period	Linear	NPIs held constant	Linear	Linear	NPIs held constant
**R0 wild strain**	Estimates in each count based on observed cases	2.4 (mean)	2.3	Transmission-specific parameters adjusted to data. R0 was not specified explicitly.	Prior in [1.6, 3.8]. Posterior defined by the model calibration in each state.	NA (due to non-mechanistic nature of the model)	Estimated from current data for current contact scores, and adjusted to pre-COVID contact scores	Variable, as we estimate intrinsic transmissibility and NPI effects through fitting of transmission rates from observed case series	NA (wild type not explicitly modeled, model initialized with delta variant with calibrated R_effective which varies among states)

Note: additional assumptions that are left at the teams' discretion are unspecified here to encapsulate current sources of scientific uncertainty (e.g., transmissibility assumptions).

Disclaimers

Any use of trade, firm, or product names is for descriptive purposes only and does not imply endorsement by the U.S. Government.
